# FunFun: ITS‐based functional annotator of fungal communities

**DOI:** 10.1002/ece3.9874

**Published:** 2023-03-08

**Authors:** Danil V. Krivonos, Dmitry N. Konanov, Elena N. Ilina

**Affiliations:** ^1^ Research Institute for Systems Biology and Medicine (RISBM) Moscow Russia

**Keywords:** bioinformatics, fungal community, fungi

## Abstract

The study of individual fungi and their communities is of great interest to modern biology because they might be both producers of useful compounds, such as antibiotics and organic acids, and pathogens of various diseases. And certain features associated with the functional capabilities of fungi are determined by differences in gene content. Information about gene content is most often taken from the results of functional annotation of the whole genome. However, in practice, whole genome sequencing of fungi is rarely performed. At the same time, usually sequence amplicons of the ITS region to identify fungal taxonomy. But in the case of amplicon sequencing there is no way to perform a functional annotation. Here, we present FunFun, the instrument that allows to evaluate the gene content of an individual fungus or mycobiome from ITS sequencing data. FunFun algorithm based on a modified *K*‐nearest neighbors algorithm. As input, the program can use ITS1, ITS2, or a full‐size ITS cluster (ITS1‐5.8S‐ITS2). FunFun was realized as a pip‐installed command line instrument and validated using a shuffle‐split approach. The developed instrument can be very useful in the fungal community comparing and estimating functional capabilities of fungi under study. Also, the program can predict with high accuracy the most variable functions.

## Graphical abstract

We have developed a tool for predicting the gene content of fungi and their communities. The program should find its application in modern biology and ecology.

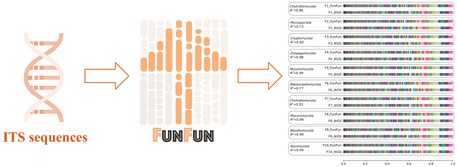

## 1 INTRODUCTION

Natural fungal communities are characterized by a high diversity of unique biochemical pathways, including the biosynthesis of antimicrobial substances, organic acids, and even toxins (Branco, 2019). At the same time, individual fungi from one community might significantly differ from each other in terms of their functional capabilities (Wisecaver et al., 2014). This is due to differences in gene content.

Functional capabilities are of great interest to modern industrial biotechnology (Chergui et al., 2021; Habibi et al., 2021; Li et al., 2020). Also, information about gene content can be useful to solve ecological problems such as the bioconversion of solid waste (Chilakamarry et al., 2022) or caffeine utilization (Zhou et al., 2018). To get a more complete picture of the functional capabilities of the fungi under study, functional annotation of whole genome assemblies is used.

Unfortunately, it is not always possible to conduct whole genome sequencing (WGS) of explored fungi. This is partly due to the high cost of WGS and some of the technical hurdles that might appear while working with eukaryotic cells. At the same time, the majority of studies of fungi are accompanied by sequencing to determine fungal taxonomy. One of the most used taxonomy markers for fungi is the ITS region (Blaalid et al., 2013; Mbareche et al., 2020; Schoch et al., 2012) The region consists of two variable subregions (ITS1 and ITS2) separated by a conserved 5.8S gene. Although the ITS sequence can provide fungal taxonomy, it does not allow for functional annotation and evaluation of gene content. In this case, a tool to predict the gene content profile for a given ITS sequence would be useful.

There are a number of tools designed to predict the biochemical features of bacterial communities using 16S sequencing data (Douglas et al., 2020; Sun et al., 2020). At the same time, functional annotation of the fungal part of the community is usually performed using WGS, since the number of precisely annotated fungi is not enough to build complex predictive models such as the algorithm implemented in PICRUST2 (Douglas et al., 2020). For this reason, the fungal part of microbial communities often remains to be neglected.

Based on a similar idea of PICRUST2, we decided to develop an algorithm that is designed to solve a task for functional annotation of fungal ITS sequences datasets. The tool is based on the assumption that the functional capabilities of fungi are in strong correlation with their genetic‐related organisms (Ke & Tsai, 2022; McLaughlin et al., 2015; Wisecaver et al., 2014). We implemented a modified *K*‐nearest neighbors (KNN) model and tested it using shuffle‐split cross‐validation on the ITS subset. The model has been implemented as a command‐line tool called FunFun.

## 2 METHODS

### 2.1 Data collection

First of all, we collected 9271 whole fungal genomes of different assembly levels from the GeneBank database. Next, we extracted ITS1‐5.8S‐ITS2 sequence fragments from the genomes via in silico PCR method with ITS1F: CTTGGTCATTTAGAGGAAGTAA and ITS4: TCCTCCGCTTATTGATATGC primers using the ipcress tool from the exonerate software package (Slater & Birney, 2005). After this stage, 6132 fungal genomes were left, which might be the result of the unsuccessful annealing of primers. Generally, in silico PCR does not guarantee the absolute correctness of extracted sequences. To ensure that the extracted fragments truly were ITS, we verified them using ITSx software, which specializes in extracting the ITS region (Bengtsson‐Palme et al., 2013). Based on the ITSx annotations, we built databases of individual ITS1 and ITS2 sequences, and full ITS1‐5.8S‐ITS2 fragments (they will be called Concatenates in the further text) belonging to 5882 fungal genomes. The collected database included representatives of 254 different families (Data S3).

### 2.2 Data processing

The genome assemblies were annotated using the Augustus tool (Keller et al., 2011). Predicted protein sequences were analyzed by the KofamKOALA tool (Aramaki et al., 2020) to obtain functional annotations. According to the results, we built gene content vectors constructed using the third level of the KoFAM hierarchy (430 vector components, presented in Data S2). Each component in this vector represents the relative abundance of the corresponding KEGG orthology group. To avoid the appearance of non‐fungal biochemical pathways, the KEGG orthology data were manually filtered by removing the 09160 Human Disease branch.

However, at this stage, we did not have absolute confidence in the correctness of the functional profiling of the protein sequences obtained using Augustus. Therefore, we decided to apply a similar approach to protein sequences based on verified data. For this, a dataset was formed from 75 fungal assemblies from the RefSeq database, for which protein sequences were available. Here, to ensure the reliability of data, we considered only the protein sequence data obtained using the Eukaryotic Annotation Propagation Pipeline (“The NCBI Eukaryotic Genome Annotation Pipeline,”, n.d.), which uses transcriptomic data to perform the functional annotation. Thereafter, for each assembly, we constructed paired functional gene content profiles, where the first profile was based on curated protein sequences data from RefSeq and the second profile was built using Augustus gene annotations. Convergence of the results was estimated using Pearson's correlation coefficient for each assembly pair independently.

To preliminary validate our method of functional content profiling, we used the t‐SNE decomposition for gene content vectors. We expected that observed clusters would be associated with fungal taxonomy, which is according to literature data (Ke & Tsai, 2022; McLaughlin et al., 2015; Wisecaver et al., 2014). Here, HDBSCAN was used for cluster identification. To estimate the convergence of observed clusters and fungal taxonomy, the Rand Index (RI) was used.

To demonstrate the correlation between fungal gene content and their lifestyle, we used a database compiled by the authors of the FunGUILD tool (Nguyen et al., 2016). To evaluate the convergence of the generated gene content clusters with lifestyle, we also applied HDBSCAN to isolate clusters, after which the clusters and lifestyles labels were compared using the Rand index.

### 2.3 Algorithm explanation

For each ITS sequence in the collected database, a relative abundance of each nucleotide k‐mer is calculated. *k* was chosen to be 5 because the k‐mers length equals 5 provides a satisfactory resolution of the fungal taxonomy, which can be seen on the dendrogram obtained by the method of hierarchical agglomerative clustering (Figure S4 in Data S1). For each sequence, we generate a k‐mer relative abundances vector with a length of 4^5^. In the further text, we will name such k‐mer abundances vectors as k‐mers vectors. Thereafter, we evaluate the cosine distance between the k‐mers vector representing the target sequence and the k‐mers vectors for each fungal reference amplicon variant (RAV) from the database. Thus, we construct a list of distances, which describe the similarity between the target sequence and each RAV from the database.

To predict the fungal gene content, the algorithm searches for KNN in the *ε* neighborhood using the generated distances list. Here, the *ε* neighborhood is the area, which limits the space where the neighbors searching will be performed. The number of neighbors not exceeding *K* and located in the area, limited by *ε* neighborhood, we call *K*_chosen_. Such an approach allows ignoring too far sequences while gene content prediction. For each *i*‐th fungi in our database we have a precalculated gene content profile *F*_*i*_ *= (f*_*1*_^*i*^ *, f*_*2*_^*i*^ *, f*_*3*_^*i*^ *, …, f*_*L*_^*i*^ *), F*_*i*_ is a vector with length *L* (*L* = 430). In *F*_*i*_, each *f*_*j*_^*i*^ value is a fraction of the individual *j*‐th function while the sum of these values is equal to 1. To calculate the functional gene content profile for the target sample we average the value of each individual function among the *K*_chosen_ neighbors.

$$F_{\text{target}}=\left(f_{1}^{\text{target}}=\frac{\sum_{i=1}^{K\text{chosen}}f_{1}^{i}}{K_{\text{chosen}}},f_{2}^{\text{target}}=\frac{\sum_{i=1}^{K\text{chosen}}f_{2}^{i}}{K_{\text{chosen}}},\ldots ,f_{L}^{\text{target}}=\frac{\sum_{i=1}^{K\text{chosen}}f_{L}^{i}}{K_{\text{chosen}}}\right)$$

where

*F*_target_—predicted gene content profile of target fungi, *K*_chosen_—number of neighbors not exceeding *K* and limited by ε neighborhood, *f*_*j*_^*i*^—fraction of *j*‐th function in the genome of *i‐*th neighbored fungi from *K*_chosen_.

**FIGURE 1:**
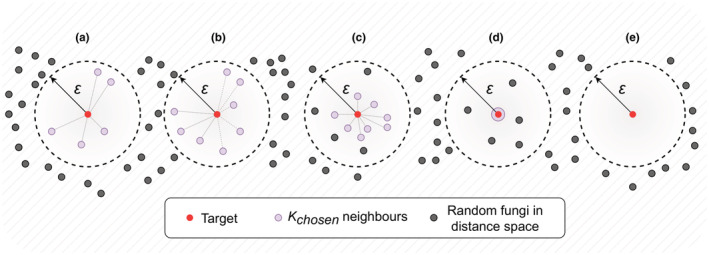
Possible cases of neighbor selection (in this example *K* = 8). (a) When the number of reference amplicon variants (RAVs) included in the ε neighborhood is less than the specified number *K*; (b) when the ε neighborhood includes exactly *K* neighbors; (c) when the number of points included in the ε neighborhood is greater than *K*; (d) when the target sequence has at least one RAV with identical k‐mers vector; and (e) when there are no neighbors in the ε neighborhood.

In cases, when the target sequence has neighbors with cosine distance to the target of 0 (that usually means the identity of ITS sequences), only these neighbors are selected for the prediction. As a result, five different cases are possible, which are visualized in Figure 1.

### 2.4 Validation

To test our algorithm, we split our database on test/train datasets and tested using shuffle‐split cross‐validation. The train/test ratio was chosen to be 80/20, with 10 shuffling iterations. So, for each shuffling epoch 1176 testing samples were used. For each epoch, the quality of prediction was estimated using a median *R* ^2^ metric between real gene content vectors, which were built using whole genome annotations, and predicted vectors which were obtained with FunFun. Validation was performed for each amplicon type (ITS1, ITS2, and Concatenate) independently.

## 3 RESULTS

### 3.1 Software description

We have developed an algorithm aimed at predicting fungal gene content using ITS sequencing data. As an input, the algorithm can use ITS1, ITS2, or full‐size ITS clusters (concatenates) in fasta format. A fasta file can contain either one sequence or several. As an output, the algorithm returns the table, where the first column is the KEGG orthology group (which we call function), and the subsequent columns correspond to predicted gene content vector components for each sample. The algorithm has been realized as a command‐line tool which we named FunFun (Fungal Functional). Originally, FunFun was developed to be capable of predicting gene content for individual fungi but this algorithm can be useful in the analysis of metagenomic analysis.

The tool has two hyperparameters *K*—the maximum number of nearest neighbors selected and *ε* that defines the area where the *K* neighbors search is performed. In the case when the tool does not return a prediction for the analyzed sequence, the user can increase the ε value. However, it should be noted here that the greater the ε value, the lower the expected prediction quality (Figure 3a). On the other hand, as we observed the number of neighbors *K* does not drastically influence the prediction quality in general but in some cases might make the prediction more robust, especially while the target sequence has a lot of close neighbors. By default, *K* is set to be 10 and ε is set to be 0.5.

### 3.2 Validation results

**FIGURE 2:**
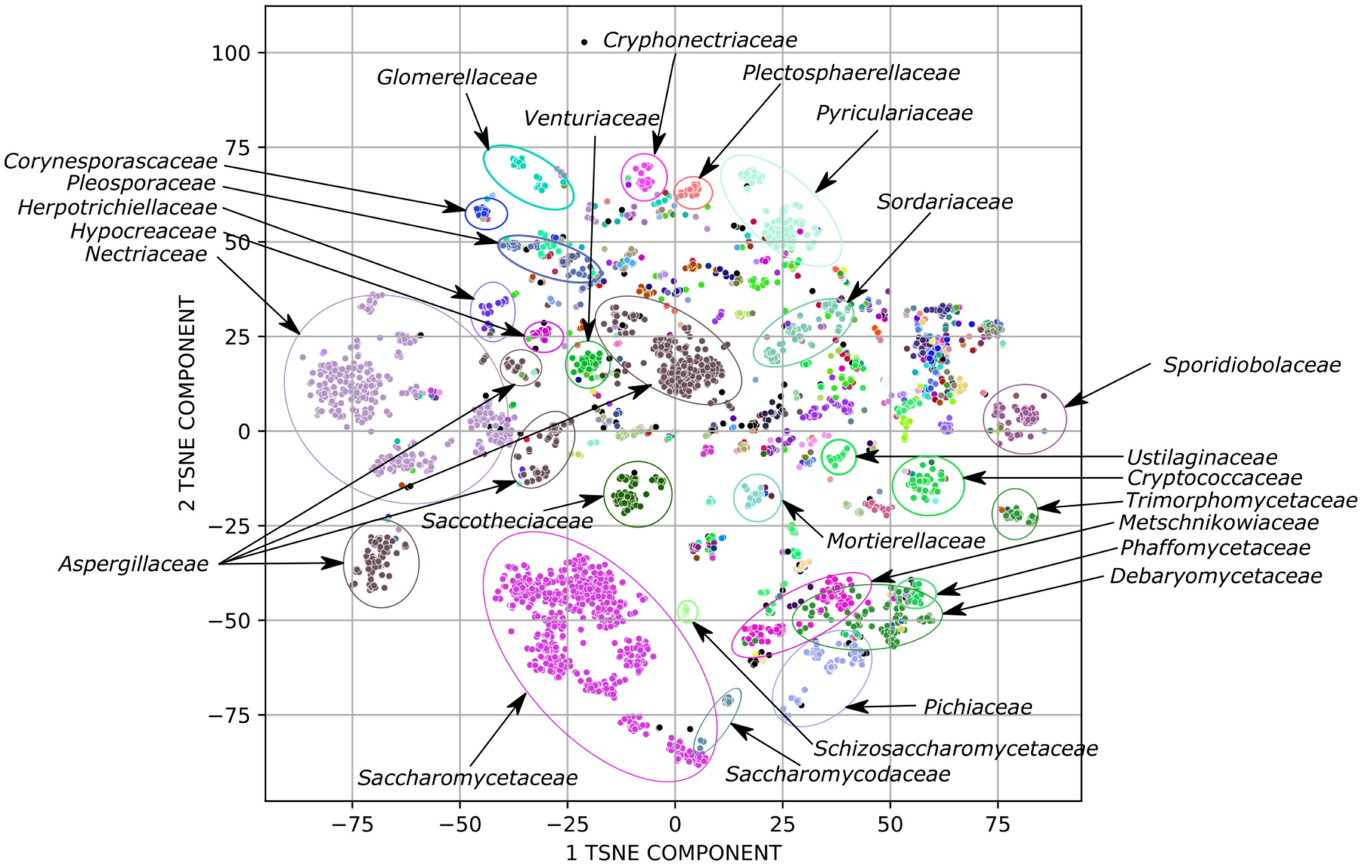
t‐SNE decomposition of fungal gene content profiles, where individual dots correspond to one sample. Each color corresponds to an individual fungal taxon (family level).

The algorithm developed is based on the assumption that fungal metabolism correlates with their taxonomy, so we expected that gene content profiles, which we calculated with our approach, also should correlate with fungal taxonomy. To verify this assumption, t‐SNE decomposition of calculated gene content vectors and clustering with the HDBSCAN algorithm (Campello et al., 2013, 2015) were performed (Figure 2). Figure 2 shows a two‐dimensional mapping of the entire space of individual functions of the gene composition; each color in this picture corresponds to the individual fungal family. Predicted clusters and NCBI taxonomy labels (family level) were compared (RI = 0.929). The largest clusters were additionally labeled in Figure 2. We have also performed a t‐SNE decomposition of 5‐mers vectors of full‐size ITS region (Figure S7 in Data S1). Additionally, we checked the relationship between clear clusters obtained on gene content vectors and corresponding full‐size ITSs (regardless of GenBank taxonomy; Figure S2 in Data S1). This test resulted in a Rand index value of 0.963 which indicates a strong correlation.

Fungal taxonomy is known to be not unambiguous (Hawksworth, 2011) which might lead to visible clustering imperfection and the presence of formal outliers for family clusters on the t‐SNE plot that can be seen in Figure 2. In addition, the t‐SNE algorithm itself tends to generate artifact clusters of dots which actually consist of different taxa (Figure S3 in Data S1). Moreover, our database is not balanced by families and some organisms may be the only members of their family (*Massarinaceae*, *Aulographaceae*, etc.), which can also affect the quality of clustering.

We also checked the validity of Augustus annotations by comparing functional profiling of Augustus annotations with corresponding RefSeq protein data. The analysis showed a good value for the Pearson correlation score (*r* = .62; Figure S1 in Data S1). Thus, the results of functional profiling obtained with Augustus are quite close to the results confirmed by experimental transcriptome data, so we decided that Augustus is suitable as a genome annotator in our method.

**FIGURE 3:**
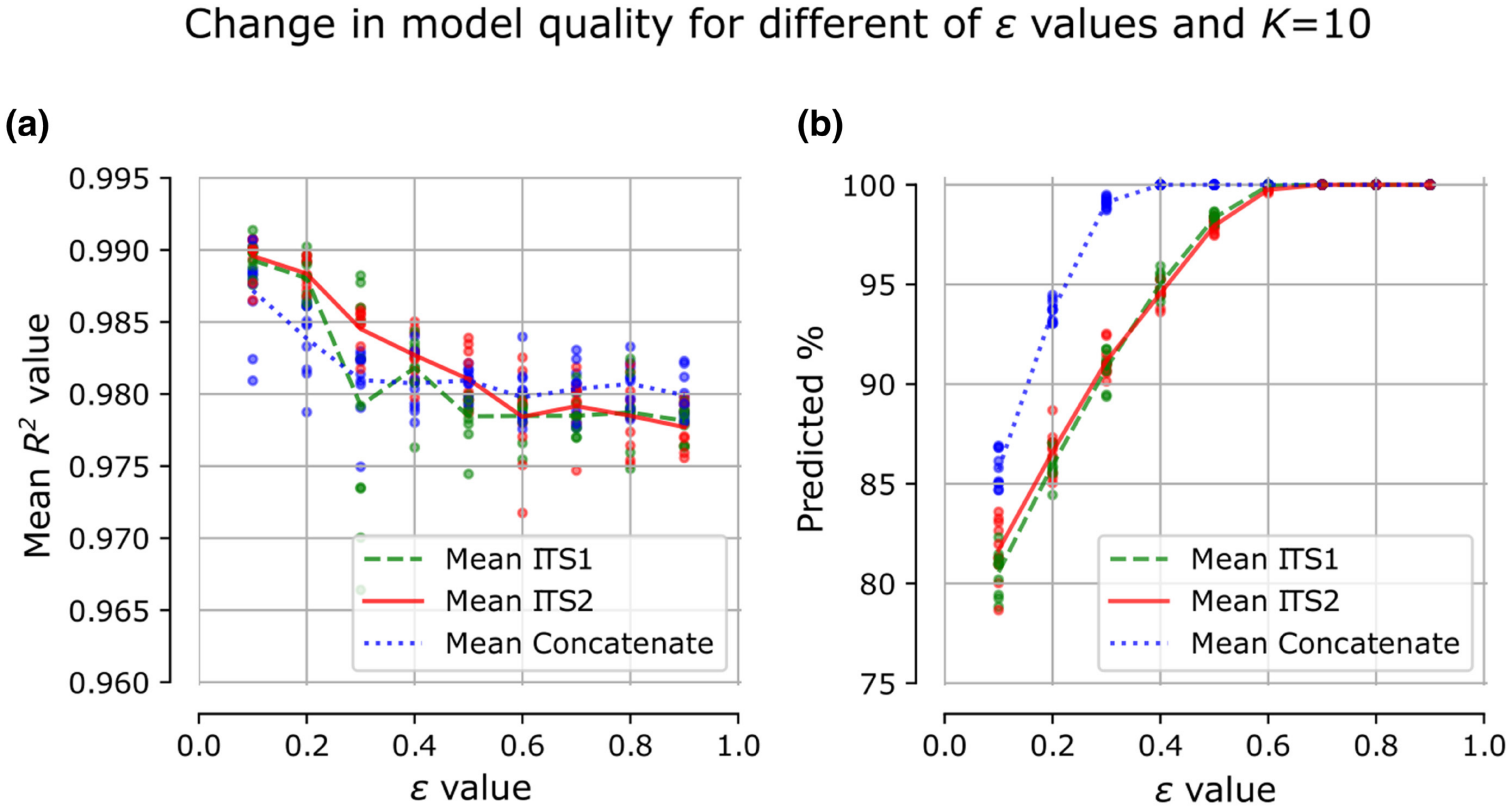
Validation results. (a) Dependence of gene content prediction quality on the *ε* value. (b) Dependence of the percentage of predictions on *ε* value (in some cases the *ε* neighborhood can include no one neighbors which does not allow to build predicted profiles with sufficient quality. Thus, the lower the *ε* value, the less the number of predicted profiles).

Using the number of neighbors of *K* = 10, we tested our algorithm with a range of different *ε* values (Figure 3). At the validation stage, we calculated the *R* ^2^ value between the gene content vector, which we built on full genome data, and the gene content vector predicted on ITS sequence using FunFun. Also, we estimated the percentage of predicted samples from the test subset (gene content is predicted only if the target ITS has at least one neighbor in the ε neighborhood).

We found that as input data, it is more efficient to pass to the algorithm the full‐size ITS sequences (concatenates). On average, the values of median *R* ^2^ were at least 0.95 regardless of the *ε* value chosen. So, we set *ε* = 0.5 and *K* = 10 as default values. If necessary, the user is able to set custom values for them when running FunFun. It should be noted that different families are presented in different ratios which definitely affects both the quality of clustering and the quality of prediction. To detect the “problem fungal families” we carried out the full FunFun pipeline on all ITS sequences in the base, and divided them by family to compare the prediction accuracy (Figure S5 in Data S1).

Quite high *R* ^2^ values might be partially explained by conservative gene functions, which are common features of all fungi. Examples of these functions are glycolysis and the citric cycle. So, we decided to additionally check how the method estimates relative abundances of the functions most variable among different fungal genomes. We chose such functions using the coefficient of variation, which is defined as the standard deviation normalized to the mean. These functions included glucosamine degradation, biosynthesis of ansamycine, toluene degradation, indole alkaloid biosynthesis, caffeine biosynthesis, and so on. We considered a function as highly variable if its coefficient of variation was greater than 10. As in the previous validation stage, we calculated the median *R* ^2^ value between real and predicted gene content vectors. In the results, we obtained median *R* ^2^ = 0.99 and the percentage of predicted equals 98.6%.

Some biochemical features are associated with fungal guilds (Liu et al., 2023; Nguyen et al., 2016; Su et al., 2023). We decided to check the convergence of guilds and gene content profiles. Guild labels were downloaded from the database used in the FUNGuild tool (Nguyen et al., 2016). Visible correlation between the guild and inferred gene content has been observed (RI = 0.84; Figure S6 in Data S1). Thus, our algorithm allows us to catch functions, characterized by fungal lifestyles.

### 3.3 Metagenome case modeling

**FIGURE 4:**
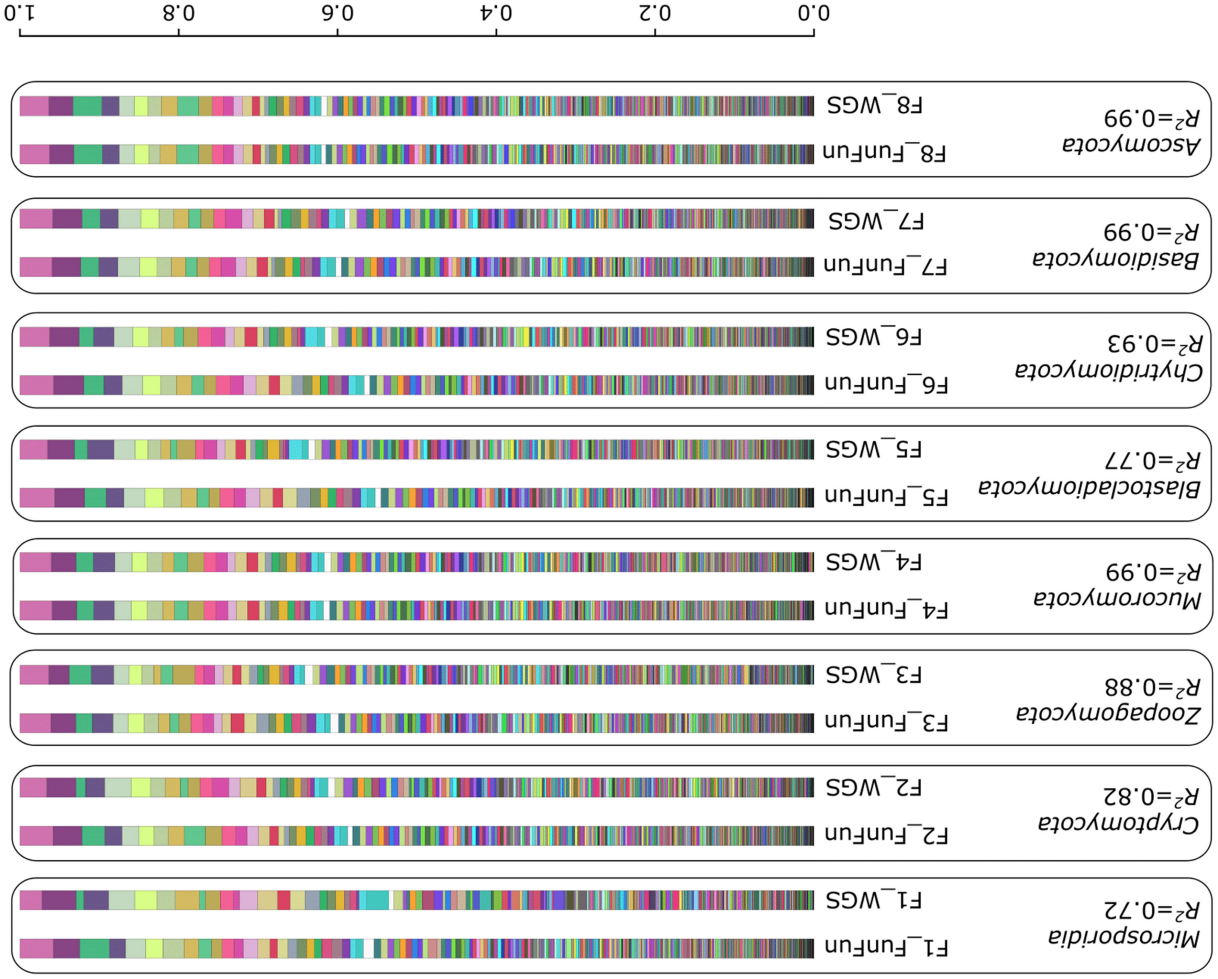
Comparison of gene content profiles based on whole genome sequence data and predicted with FunFun for each ITS from the simulated metagenome. For each pair, the lower bar represents the actual fractions of corresponding functions in the genome, while the upper bar shows function fractions predicted with FunFun.

In our view, the metagenomic analysis should be carried out after obtaining consensus assemblies of ITS sequences, after which the resulting multi‐fasta can be processed by FunFun. To simulate this case, we took random sequences from our database for eight different divisions and predicted gene content profiles for them using our approach and compared the convergence of the predicted and real profiles using *R* ^2^ (Figure 4). For prediction, we removed the predicted sequence from our database. Note that our algorithm does not use relative abundances of the fungi in the community, but returns just profiles for individual ITS consensus sequences since it is rather difficult to precisely estimate fungal composition in the sample due to the lack of reliable and reproducible methods to analyze mycobiome data (Martinsen et al., 2021; Weaver et al., 2019).

## 4 CONCLUSION

Fungi being an essential part of microbial communities often remain neglected during metagenomic studies. In this work, we tried to develop an algorithm that would be capable of predicting fungal gene content based not on whole genome sequencing but using amplicon sequencing data, which is significantly less expensive. At the same time, there are no such tools for predicting the functional capability by the marker sequence for fungi.

Thus, we have developed FunFun, a novel tool designed for the estimation of fungal functional capability based on ITS amplicon sequencing data. It can be useful for the estimation of fungal gene content in individual fungi as well as in mycobiome. We should note that our approach has certain limitations while analyzing fungal ITS sequences which do not have close RAVs presented in the collected database. However, the authors hope that, in the future, the gradual addition of new genomes to the FunFun database will partially solve this issue. In this initial release, the database was collected from GenBank only and did not include the manually curated genomes from Mycocosm (Grigoriev et al., 2014) which we are planning to reannotate and add to FunFun in further releases.

We have shown that our approach allows us to predict not only the common functions such as a citric cycle or glycolysis but also the most varied. It might be helpful for researchers, who want to estimate the gene content of the investigated fungi.

## AUTHOR CONTRIBUTIONS

**Danil V. Krivonos:** Conceptualization (equal); data curation (equal); formal analysis (equal); software (lead); visualization (lead); writing – original draft (equal). **Dmitry N. Konanov:** Conceptualization (equal); formal analysis (equal); methodology (equal); writing – original draft (equal). **Elena N. Ilina:** Writing – review and editing (equal).

## CONFLICT OF INTEREST STATEMENT

The authors declare no conflict of interest.

## Supporting information


Data S1.

Data S2.

Data S3.

## Data Availability

FunFun can be installed via pip and can be downloaded from https://github.com/DanilKrivonos/FunFun.

## References

[ece39874-bib-0001] Aramaki, T. , Blanc‐Mathieu, R. , Endo, H. , Ohkubo, K. , Kanehisa, M. , Goto, S. , & Ogata, H. (2020). KofamKOALA: KEGG Ortholog assignment based on profile HMM and adaptive score threshold. Bioinformatics, 36, 2251–2252. 10.1093/bioinformatics/btz859 31742321 PMC7141845

[ece39874-bib-0002] Bengtsson‐Palme, J. , Ryberg, M. , Hartmann, M. , Branco, S. , Wang, Z. , Godhe, A. , De Wit, P. , Sánchez‐García, M. , Ebersberger, I. , de Sousa, F. , Amend, A. , Jumpponen, A. , Unterseher, M. , Kristiansson, E. , Abarenkov, K. , Bertrand, Y. J. K. , Sanli, K. , Eriksson, K. M. , Vik, U. , … Nilsson, R. H. (2013). Improved software detection and extraction of ITS1 and ITS2 from ribosomal ITS sequences of fungi and other eukaryotes for analysis of environmental sequencing data. Methods in Ecology and Evolution, 4, 914–919. 10.1111/2041-210X.12073

[ece39874-bib-0003] Blaalid, R. , Kumar, S. , Nilsson, R. H. , Abarenkov, K. , Kirk, P. M. , & Kauserud, H. (2013). ITS1 versus ITS2 as DNA metabarcodes for fungi. Molecular Ecology Resources, 13, 218–224. 10.1111/1755-0998.12065 23350562

[ece39874-bib-0004] Branco, S. (2019). Fungal diversity from communities to genes. Fungal Biology Reviews, 33, 225–237. 10.1016/j.fbr.2019.06.003

[ece39874-bib-0005] Campello, R. J. G. B. , Moulavi, D. , & Sander, J. (2013). Density‐based clustering based on hierarchical density estimates. In J. Pei , V. S. Tseng , L. Cao , H. Motoda , & G. Xu (Eds.), Advances in knowledge discovery and data mining, lecture notes in computer science (pp. 160–172). Springer Berlin Heidelberg. 10.1007/978-3-642-37456-2_14

[ece39874-bib-0006] Campello, R. J. G. B. , Moulavi, D. , Zimek, A. , & Sander, J. (2015). Hierarchical density estimates for data clustering, visualization, and outlier detection. ACM Transactions on Knowledge Discovery from Data, 10, 1–51. 10.1145/2733381

[ece39874-bib-0007] Chergui, D. , Akretche‐Kelfat, S. , Lamoudi, L. , Al‐Rshaidat, M. , Boudjelal, F. , & Ait‐Amar, H. (2021). Optimization of citric acid production by aspergillus Niger using two downgraded Algerian date varieties. Saudi Journal of Biological Sciences, 28, 7134–7141. 10.1016/j.sjbs.2021.08.013 34867016 PMC8626340

[ece39874-bib-0008] Chilakamarry, C. R. , Mimi Sakinah, A. M. , Zularisam, A. W. , Sirohi, R. , Khilji, I. A. , Ahmad, N. , & Pandey, A. (2022). Advances in solid‐state fermentation for bioconversion of agricultural wastes to value‐added products: Opportunities and challenges. Bioresource Technology, 343, 126065. 10.1016/j.biortech.2021.126065 34624472

[ece39874-bib-0009] Douglas, G. M. , Maffei, V. J. , Zaneveld, J. R. , Yurgel, S. N. , Brown, J. R. , Taylor, C. M. , Huttenhower, C. , & Langille, M. G. I. (2020). PICRUSt2 for prediction of metagenome functions. Nature Biotechnology, 38, 685–688. 10.1038/s41587-020-0548-6 PMC7365738 32483366

[ece39874-bib-0010] Grigoriev, I. V. , Nikitin, R. , Haridas, S. , Kuo, A. , Ohm, R. , Otillar, R. , Riley, R. , Salamov, A. , Zhao, X. , Korzeniewski, F. , Smirnova, T. , Nordberg, H. , Dubchak, I. , & Shabalov, I. (2014). MycoCosm portal: Gearing up for 1000 fungal genomes. Nucleic Acids Research, 42(D1), D699–D704. 10.1093/nar/gkt1183 24297253 PMC3965089

[ece39874-bib-0011] Habibi, A. , Karami, S. , Varmira, K. , & Hadadi, M. (2021). Key parameters optimization of chitosan production from aspergillus terreus using apple waste extract as sole carbon source. Bioprocess and Biosystems Engineering, 44, 283–295. 10.1007/s00449-020-02441-2 32959145

[ece39874-bib-0012] Hawksworth, D. L. (2011). A new dawn for the naming of fungi: Impacts of decisions made in Melbourne in July 2011 on the future publication and regulation of fungal names. IMA Fungus, 2, 155–162. 10.5598/imafungus.2011.02.02.06 22679600 PMC3359813

[ece39874-bib-0013] Ke, H.‐M. , & Tsai, I. J. (2022). Understanding and using fungal bioluminescence – Recent progress and future perspectives. Current Opinion in Green and Sustainable Chemistry, 33, 100570. 10.1016/j.cogsc.2021.100570

[ece39874-bib-0014] Keller, O. , Kollmar, M. , Stanke, M. , & Waack, S. (2011). A novel hybrid gene prediction method employing protein multiple sequence alignments. Bioinformatics, 27, 757–763. 10.1093/bioinformatics/btr010 21216780

[ece39874-bib-0015] Li, Y. , Chen, Y. , Tian, X. , & Chu, J. (2020). Advances in sophorolipid‐producing strain performance improvement and fermentation optimization technology. Applied Microbiology and Biotechnology, 104, 10325–10337. 10.1007/s00253-020-10964-7 33097965

[ece39874-bib-0016] Liu, Y. , Dong, L. , Zhang, H. , Deng, Y. , Hu, B. , & Wang, W. (2023). Distinct roles of bacteria and fungi in mediating soil extracellular enzymes under long‐term nitrogen deposition in temperate plantations. Forest Ecology and Management, 529, 120658. 10.1016/j.foreco.2022.120658

[ece39874-bib-0017] Martinsen, E. M. H. , Eagan, T. M. L. , Leiten, E. O. , Haaland, I. , Husebø, G. R. , Knudsen, K. S. , Drengenes, C. , Sanseverino, W. , Paytuví‐Gallart, A. , & Nielsen, R. (2021). The pulmonary mycobiome‐a study of subjects with and without chronic obstructive pulmonary disease. PLoS ONE, 16, e0248967. 10.1371/journal.pone.0248967 33826639 PMC8026037

[ece39874-bib-0018] Mbareche, H. , Veillette, M. , Bilodeau, G. , & Duchaine, C. (2020). Comparison of the performance of ITS1 and ITS2 as barcodes in amplicon‐based sequencing of bioaerosols. PeerJ, 8, e8523. 10.7717/peerj.8523 32110484 PMC7032056

[ece39874-bib-0019] McLaughlin, D. J. , Kumar, T. K. A. , Blackwell, M. , Letcher, P. M. , & Roberson, R. W. (2015). 9 Subcellular structure and biochemical characters in fungal phylogeny. In D. J. McLaughlin & J. W. Spatafora (Eds.), Systematics and evolution: Part B, the Mycota (pp. 229–258). Springer. 10.1007/978-3-662-46011-5_9

[ece39874-bib-0020] Nguyen, N. H. , Song, Z. , Bates, S. T. , Branco, S. , Tedersoo, L. , Menke, J. , Schilling, J. S. , & Kennedy, P. G. (2016). FUNGuild: An open annotation tool for parsing fungal community datasets by ecological guild. Fungal Ecology, 20, 241–248. 10.1016/j.funeco.2015.06.006

[ece39874-bib-0021] Schoch, C. L. , Seifert, K. A. , Huhndorf, S. , Robert, V. , Spouge, J. L. , Levesque, C. A. , Chen, W. , & Fungal Barcoding Consortium, Fungal Barcoding Consortium Author List . (2012). Nuclear ribosomal internal transcribed spacer (ITS) region as a universal DNA barcode marker for Fungi. Proceedings of the National Academy of Sciences of the United States of America, 109, 6241–6246. 10.1073/pnas.1117018109 22454494 PMC3341068

[ece39874-bib-0022] Slater, G. S. C. , & Birney, E. (2005). Automated generation of heuristics for biological sequence comparison. BMC Bioinformatics, 6, 31. 10.1186/1471-2105-6-31 15713233 PMC553969

[ece39874-bib-0023] Su, J. , Ji, W. , Sun, X. , Wang, H. , Kang, Y. , & Yao, B. (2023). Effects of different management practices on soil microbial community structure and function in alpine grassland. Journal of Environmental Management, 327, 116859. 10.1016/j.jenvman.2022.116859 36450164

[ece39874-bib-0024] Sun, S. , Jones, R. B. , & Fodor, A. A. (2020). Inference‐based accuracy of metagenome prediction tools varies across sample types and functional categories. Microbiome, 8, 46. 10.1186/s40168-020-00815-y 32241293 PMC7118876

[ece39874-bib-0025] The NCBI . (n.d.). Eukaryotic genome annotation pipeline [WWW document] . Retrieved from July 18, 2022 from https://www.ncbi.nlm.nih.gov/genome/annotation_euk/process/#references

[ece39874-bib-0026] Weaver, D. , Gago, S. , Bromley, M. , & Bowyer, P. (2019). The human lung Mycobiome in chronic respiratory disease: Limitations of methods and our current understanding. Current Fungal Infection Reports, 13, 109–119. 10.1007/s12281-019-00347-5

[ece39874-bib-0027] Wisecaver, J. H. , Slot, J. C. , & Rokas, A. (2014). The evolution of fungal metabolic pathways. PLoS Genetics, 10, e1004816. 10.1371/journal.pgen.1004816 25474404 PMC4256263

[ece39874-bib-0028] Zhou, B. , Ma, C. , Wang, H. , & Xia, T. (2018). Biodegradation of caffeine by whole cells of tea‐derived fungi aspergillus sydowii, aspergillus Niger and optimization for caffeine degradation. BMC Microbiology, 18, 53. 10.1186/s12866-018-1194-8 29866035 PMC5987490

