# Supplementary material for: FunFun: ITS‐based functional annotator of fungal communities
Source: Ecol Evol. 2023 Mar 8;13(3):e9874. doi: 10.1002/ece3.9874 (PMC9994472; doi:10.1002/ece3.9874)
Supplement: Supplementary file 1 — Data S1. [file ECE3-13-e9874-s001.docx]

SUPPLEMENTARY 1 Figures


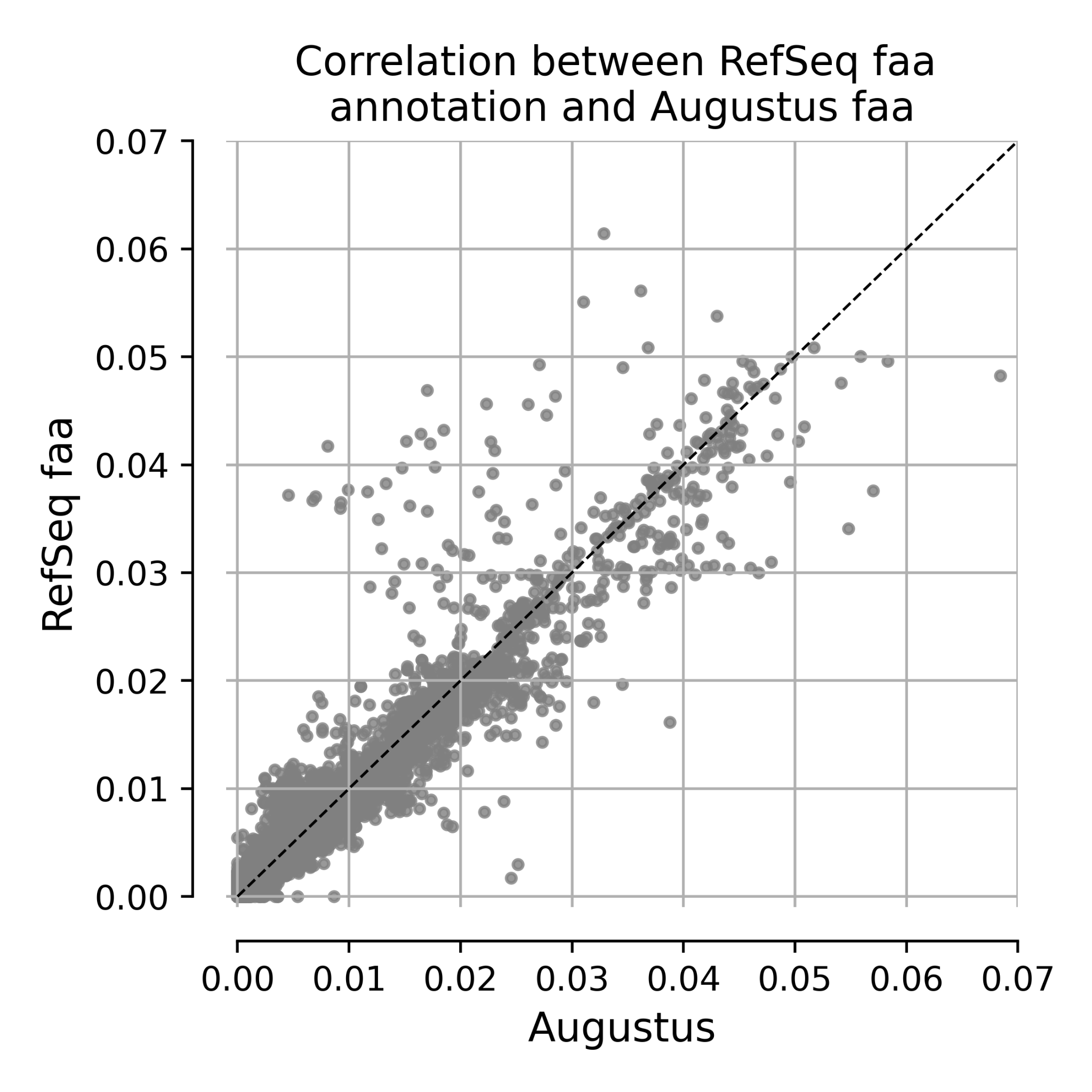


FIGURE 1 Convergence of kofam annotation for RefSeq faa file and Augustus faa. Pearson correlation coefficient is 0.62.


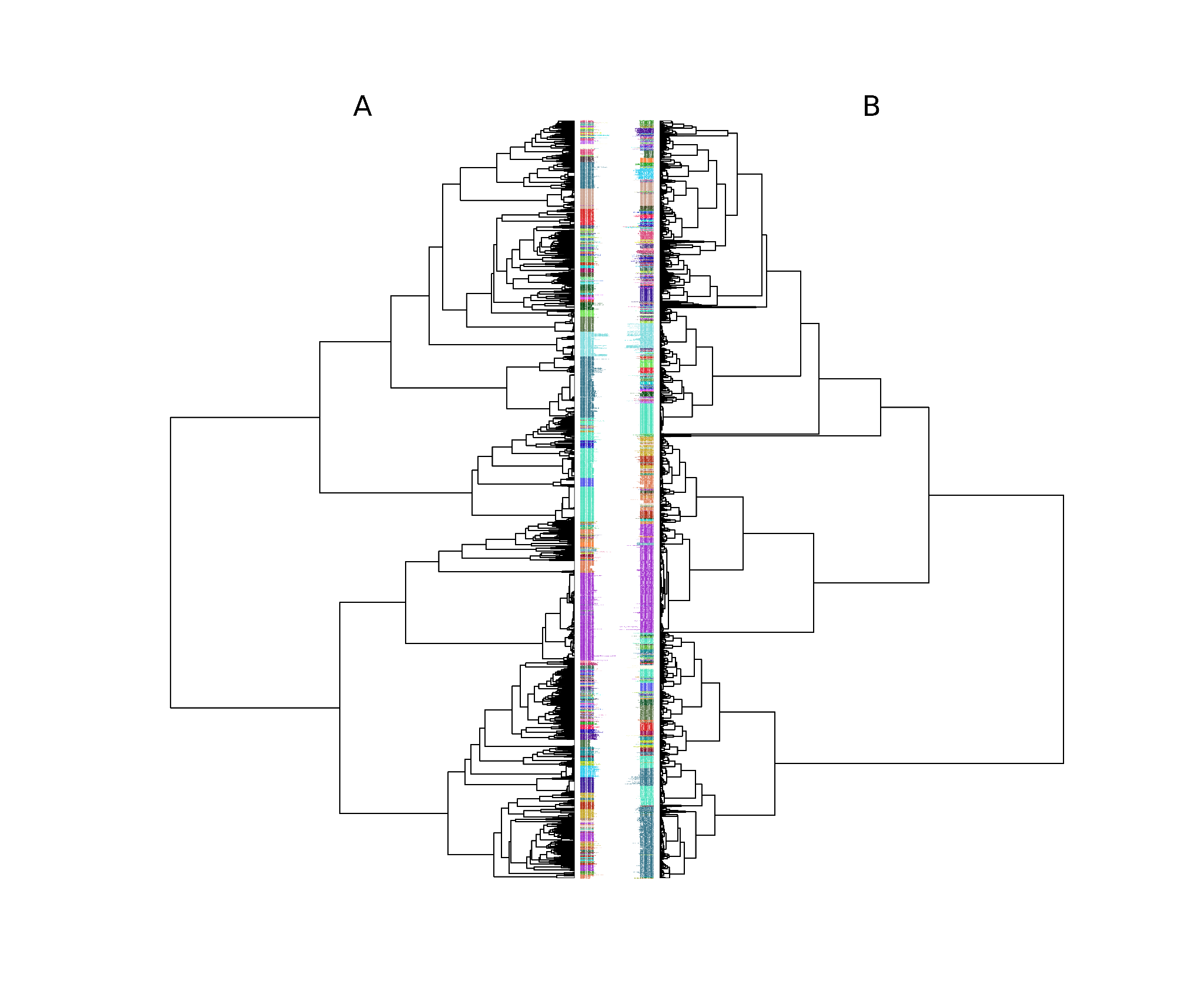


FIGURE 2 Dendrograms were built using agglomerative clustering for 5-mers vectors of full size ITS cluster (A) and gene content profiles (B). Each fungal family is marked with unique color. This analysis resulted with a Rand index value of 0.963.


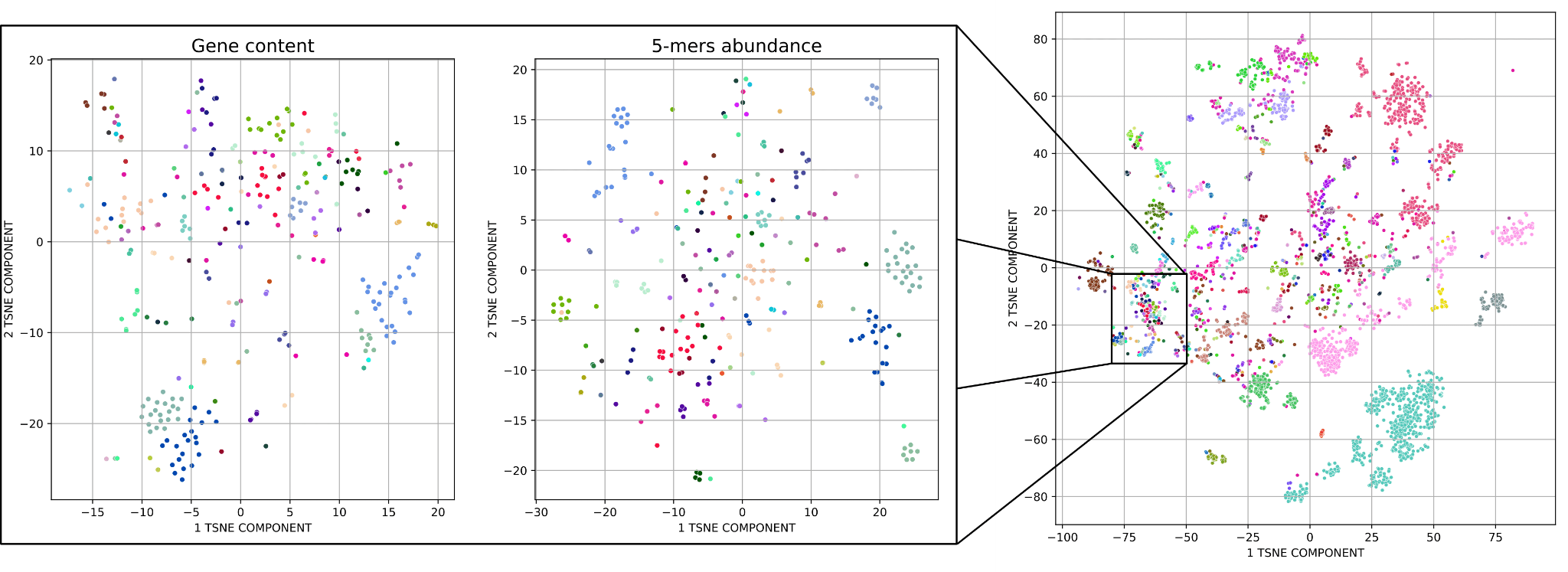


FIGURE 3 Comparison local t-SNE decomposition of gene content profiles and 5-mers vectors of full size ITS sequence. Here were chosen location, which include fungi from different families.


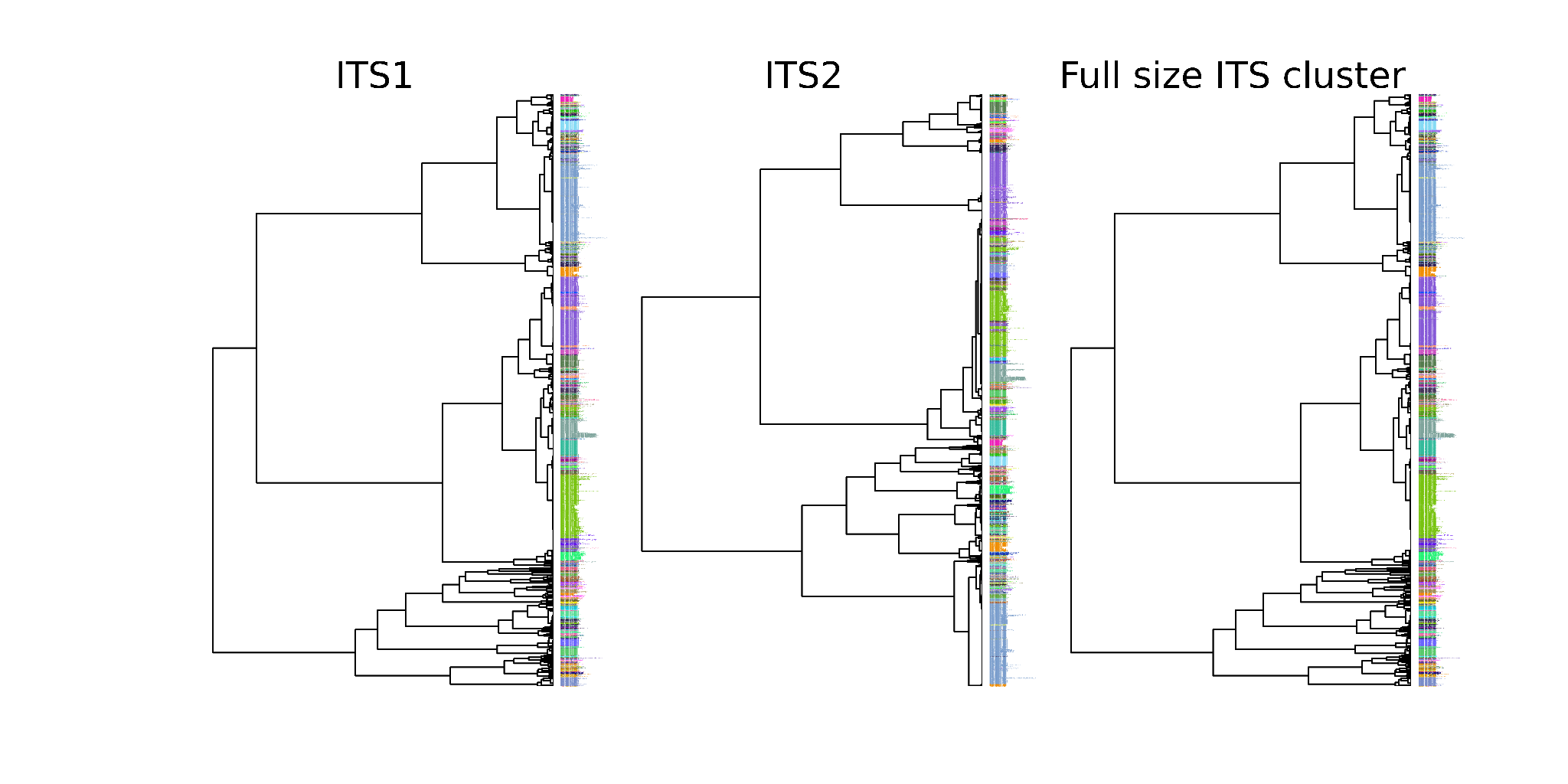


FIGURE 4 Hierarchical agglomerative clustering of 5-mers vectors for different types of ITS sequences (ITS1, ITS2 and ITS1+5.8S+ITS2). Each fungal family marked with unique color.


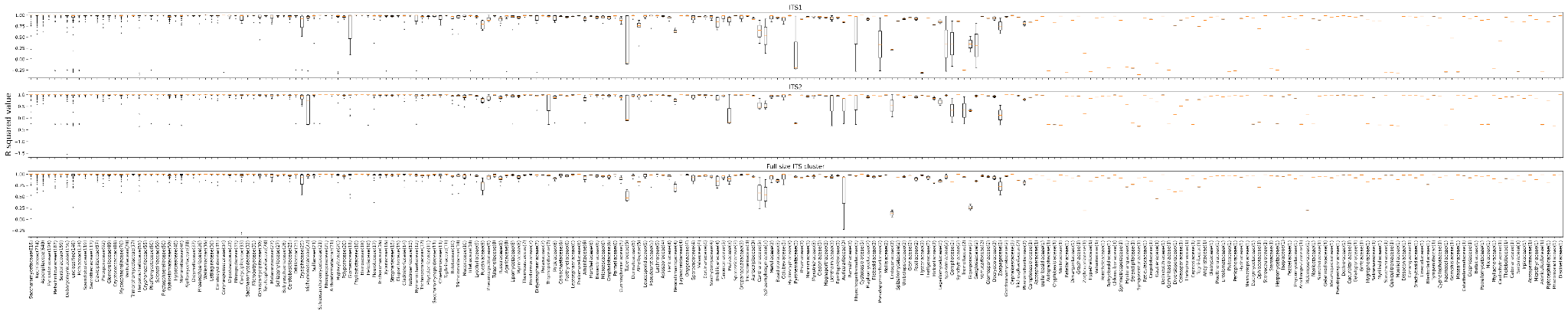


FIGURE 5 R^2^ value for different fungal families for different ITS sequence type.


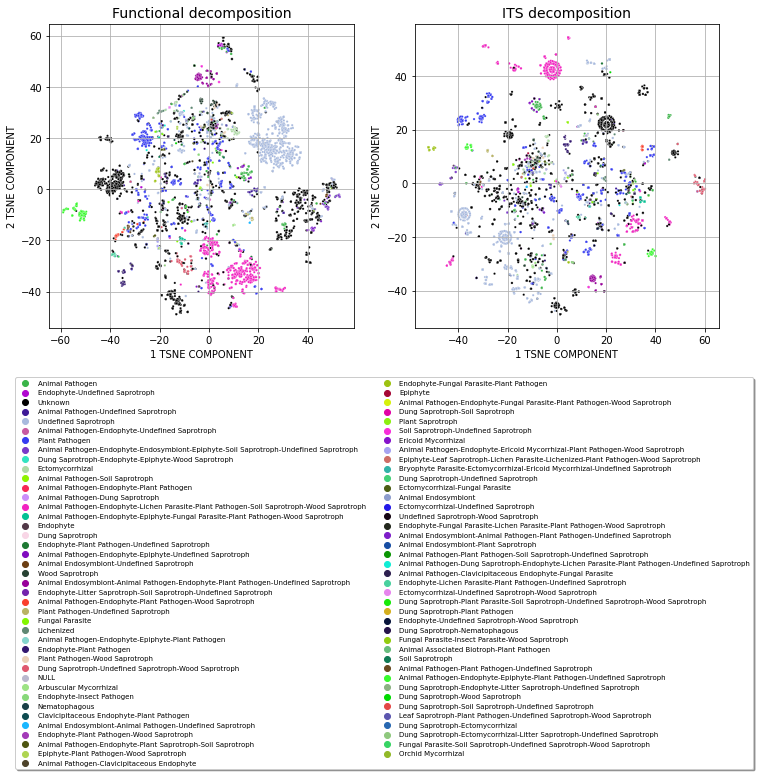


FIGURE 6 t-SNE decomposition of fungal gene content profiles (left) and ITS 5-mers signature (right). Here individual dots correspond to one sample. Unique color corresponds to an individual fungal habitat niche.


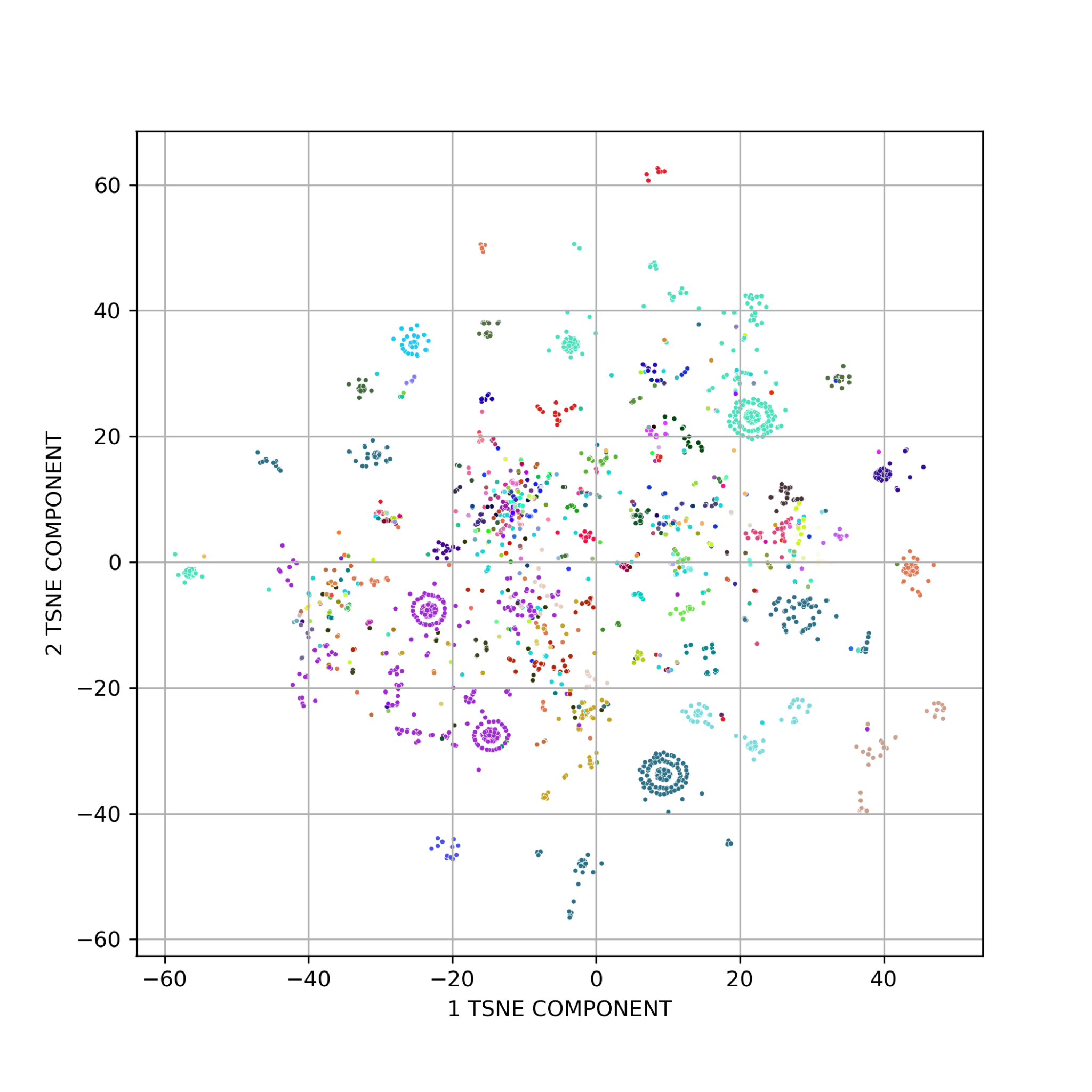


FIGURE 7 t-SNE decomposition of 5-mers vectors for full size ITS region. Here individual dots correspond to one sample. Each fungal family marked with unique color. (Rand Index=0.92)
