# Supplementary material for: FunFun: ITS‐based functional annotator of fungal communities
Source: Ecol Evol. 2023 Mar 8;13(3):e9874. doi: 10.1002/ece3.9874 (PMC9994472; doi:10.1002/ece3.9874)
Supplement: Supplementary file 3 — Data S3. [file ECE3-13-e9874-s002.pdf]

Kingdom	Subkingdom	Division	Unranked	Subdivision	Unranked	Class	Subclass	Order	Suborder	Family	No of species in FunFun database	Median R2 for		
												ITS1	ITS2	Full size ITS
Fungi	Dikarya	Ascomycota	Saccharomyceta	Pezizomycotina	Leotiomyceta	Dothideomycetes	Pleosporomycetidae	Pleosporales	Massarineae	Massarinaceae	1	-0.29	0.96	0.98
										Didymosphaeriaceae	7	0.98	0.95	0.98
										Amniculicolaceae	1	-0.27	-0.27	0.99
										Sporormiaceae	2	0.35	0.97	0.94
										Pleosporineae	174	1.0	1.0	1.0
										Corynesporascaceae	34	1.0	1.0	1.0
										Dothidotthiaceae	1	0.98	0.98	0.99
										Pleosporales incertae sedis	4	0.88	0.96	0.97
								Aulographales		Aulographaceae	1	-0.26	-0.26	0.89
								Mytilinidiales		Mytilinidiaceae	1	0.88	-0.29	0.9
								Venturiales		Sympoventuriaceae	1	-0.34	-0.34	0.91
										Venturiaceae	70	1.0	1.0	1.0
								Hysteriales		Hysteriaceae	2	-0.3	0.98	0.97
								Pleosporomycetidae incertae sedis		Gloniaceae	2	0.94	0.96	0.97
							Dothideomycetidae	Myriangiales		Elsinoaceae	18	1.0	1.0	1.0
								Mycosphaerellales		Mycosphaerellaceae	76	0.99	0.99	0.98
										Dissoconiaceae	1	0.97	0.98	0.98
										Teratosphaeriaceae	28	1.0	1.0	1.0
								Dothideales		Dothioraceae	1	0.99	0.98	0.98
										Saccotheciaceae	117	1.0	1.0	1.0
								Capnodiales		Capnodiaceae	1	0.97	-0.27	0.97
										Capnodiales incertae sedis	1	0.98	0.98	0.97
								Cladosporiales		Cladosporiaceae	14	0.99	0.99	0.99
							Dothideomycetes incertae sedis	Acrospermales		Venturiaceae	70	1.0	1.0	1.0
								Microthyriales		Microthyriaceae	1	0.9	0.85	0.88
								Valsariales		Valsariaceae	1	0.93	0.88	0.93
								Botryosphaeriales		Phyllostictaceae	18	1.0	1.0	1.0
										Botryosphaeriaceae	26	1.0	1.0	1.0
								Lineolatales		Lineolataceae	1	0.98	0.92	0.98
								Patellariales		Patellariaceae	1	-0.29	0.94	0.97
								Trypetheliales		Trypetheliaceae	1	-0.28	0.83	0.85
										Pseudoperisporiaceae	1	0.97	0.97	0.97
						Sordariomyceta	Leotiomycetes	Erysiphales		Erysiphaceae	25	0.92	0.93	0.93
								Leotiales		Tympanidaceae	1	0.97	0.97	0.99
								Thelebolales		Thelebolaceae	1	0.95	0.91	0.9
								Helotiales		Rutstroemiaceae	12	1.0	1.0	1.0
										Drepanopezizaceae	3	0.99	1.0	0.98
										Cenangiaceae	1	0.98	0.97	0.98
										Lachnaceae	5	0.98	0.99	0.99
										Discinellaceae	1	0.98	0.99	0.99
										Arachnopezizaceae	1	0.98	-0.26	0.97
										Sclerotiniaceae	50	1.0	1.0	1.0
										Mollisiaceae	2	0.98	0.99	0.99
										Chlorociboriaceae	1	0.98	0.99	0.98
										Helotiaceae	18	1.0	1.0	1.0
										Gelatinodiscaceae	1	0.98	0.98	0.95
										Hyaloscyphaceae	3	0.99	0.99	0.99
										Dermateaceae	5	0.99	0.94	0.97
										Pezizellaceae	1	0.97	0.99	0.98
										Ploettnerulaceae	1	0.98	0.98	0.96
										Helotiales incertae sedis	9	0.99	0.98	0.98
								Leotiomycetes incertae sedis		Myxotrichaceae	3	0.98	0.98	0.97
										Pseudeurotiaceae	5	0.98	0.97	0.98
										Chaetomiaceae	11	0.98	0.98	0.97
							Laboulbeniomycetes	Herpomycetales		Herpomycetaceae	1	-0.24	-0.24	0.96
							ordariomycete / Hypocreomycetidae	Microascales		Graphiaceae	1	0.98	0.93	0.96
										Ceratocystidaceae	35	0.99	0.99	1.0
										Microascaceae	6	0.96	0.97	0.96
										Gondwanamycetaceae	2	0.96	0.99	0.96
								Glomerellales		Reticulascaceae	1	0.93	0.95	0.93
										Plectosphaerellaceae	50	1.0	1.0	1.0
										Glomerellaceae	89	1.0	1.0	1.0
								Hypocreales		Ophiocordycipitaceae	30	0.98	0.98	0.98
										Cordycipitaceae	33	0.98	0.98	0.98
										Clavicipitaceae	97	1.0	1.0	1.0
										Hypocreaceae	48	1.0	1.0	1.0
										Nectriaceae	714	1.0	1.0	1.0
										Stachybotryaceae	4	0.97	0.96	0.95
										Bionectriaceae	6	0.95	0.98	0.97
										Bionectriaceae	6	0.95	0.98	0.97
							Xylariomycetidae	Xylariales		Hypoxylaceae	20	0.99	0.99	0.99
										Sporocadaceae	4	0.95	0.92	0.92
										Diatrypaceae	1	0.95	0.94	0.95
										Apiosporaceae	4	0.98	0.98	0.98
										Xylariaceae	16	0.99	0.99	0.99
										Xylariales incertae sedis	12	0.99	0.99	0.99
							Sordariomycetidae	Sordariales		Chaetomiaceae	11	0.98	0.98	0.97
										Sordariaceae	159	1.0	1.0	1.0
										Sordariales incertae sedis	1	0.97	0.97	0.97
								Coniochaetales		Coniochaetaceae	6	0.97	0.99	0.99
								Magnaporthales		Ophioceraceae	1	0.7	-0.17	0.71
										Magnaporthaceae	3	0.95	0.96	0.95
										Pyriculariaceae	194	1.0	1.0	1.0
								Togniniales		Togniniaceae	1	0.75	0.79	0.72
								Phyllachorales		Phyllachoraceae	1	-0.22	0.97	0.9
								Diaporthales		Diaporthaceae	15	0.99	0.99	0.99
										Valsaceae	4	0.96	0.98	0.96
										Juglanconidaceae	4	0.99	0.99	0.99
										Gnomoniaceae	3	0.99	0.98	0.96
										Cryphonectriaceae	55	1.0	1.0	1.0
								Ophiostomatales		Ophiostomataceae	50	0.97	0.98	0.98
							Sordariomycetes incertae sedis	Trichosphaeriales		Trichosphaeriaceae	2	0.95	0.96	0.86
						Eurotiomycetes	Eurotiomycetidae	Onygenales		Nannizziopsiaceae	1	0.97	0.97	0.97
										Ascosphaeraceae	1	0.88	0.99	0.99
										Ajellomycetaceae	38	1.0	1.0	1.0
										Onygenaceae	4	0.98	0.99	0.99
										Arthrodermataceae	21	1.0	1.0	1.0
										Onygenales incertae sedis	22	1.0	1.0	1.0
								Eurotiales		Trichocomaceae	16	0.99	0.99	0.99
										Thermoascaceae	7	0.99	0.99	0.99
										Aspergillaceae	649	1.0	1.0	1.0
										Elaphomycetaceae	2	0.91	0.91	0.96
							Chaetothyriomycetidae	Phaeomoniellales		Phaeomoniellaceae	1	0.97	-0.32	0.98
								Verrucariales		Verrucariaceae	2	0.97	0.99	0.96
								Chaetothyriales		Trichomeriaceae	3	0.99	1.0	0.99
										Herpotrichiellaceae	55	1.0	1.0	1.0
										Cyphellophoraceae	1	0.93	0.93	0.93
						Geoglossomycetes		Geoglossales		Geoglossaceae	2	0.31	0.95	0.85
						Lecanoromycetes					19	0.98	0.98	0.99
						Xylonomycetes					1	0.91	0.99	0.95
					Pezizomycetes			Pezizales		Tuberaceae	5	-0.07	-0.07	0.47
										Pezizaceae	3	0.93	-0.16	0.9
										Rhizinaceae	3	0.91	0.98	0.92
										Pyronemataceae	3	-0.2	-0.24	0.93
										Morchellaceae	6	0.98	1.0	0.98
					Orbiliomycetes			Orbiliales		Orbiliaceae	25	1.0	1.0	1.0
				Saccharomycotina						Saccharomycetes	1496	1.0	1.0	1.0
			Taphrinomycotina	Taphrinomycotina						Pneumocystidomycetes	9	0.89	0.89	0.96
										Taphrinomycetes	17	1.0	1.0	0.99
										Neolectomycetes	1	0.92	-0.24	0.92
										Schizosaccharomycetes	21	1.0	1.0	1.0
			unclassified Ascomycota								1	0.92	0.98	0.92
		Basidiomycota	Blastocladiomycota incertae sedis							Blastocladiomycetes	2	0.81	0.85	0.83
			Pucciniomycotina							Mixiomycetes	1	0.9	-0.26	0.96
										Pucciniomycetes	15	0.73	0.68	0.65
										Cystobasidiomycetes	4	1.0	1.0	1.0
										Microbotryomycetes	156	0.99	1.0	1.0
			Agaricomycotina							Tremellomycetes	174	1.0	1.0	1.0
										Agaricomycetes	336	0.97	0.97	0.97
										Dacrymycetes	1	0.94	-0.26	0.97
			Wallemiomycotina							Wallemiomycetes	36	1.0	1.0	1.0
										Geminibasidiomycetes	1	-0.25	-0.25	0.67
			Ustilaginomycotina							Malasseziomycetes	28	0.98	0.98	0.97
										Exobasidiomycetes	15	0.99	0.98	0.99
										Moniliellomycetes	1	-0.32	-0.32	0.88
										Ustilaginomycetes	38	1.0	1.0	1.0
	Fungi incertae sedi	Muco	Glomeromycotina							Glomeromycetes	21	0.96	0.98	0.99
			Mortierellomycotina							Mortierellomycetes	46	0.99	0.99	0.99
		Blastocladion	Mucoromycotina							Endogonomycetes	3	0.23	0.06	0.2
										Umbelopsidomycetes	2	0.91	0.9	0.94
										Mucoromycetes	53	0.99	0.98	1.0
			Blastocladiomycota incertae sedis							Blastocladiomycetes	2	0.81	0.85	0.83
		Mi	Apansporoblastina							Nosematidae	1	0.72	0.72	0.72
		Chytr	Chytridiomycota incertae sedis							Chytridiomycetes	11	0.87	0.99	0.93
										Neocallimastigomycetes	4	0.63	0.77	0.69
		Zoopagomycota	Zoopagomycotina							Zoopagomycetes	3	0.85	0.54	0.81
			Kickxellomycotina							Harpellomycetes	2	0.85	0.69	0.84
										Dimargaritomycetes	1	0.88	0.92	0.91
										Kickxellomycetes	2	0.78	0.84	0.8
			Entomophthoromycotina							Basidiobolomycetes	3	0.95	0.96	0.86
										Entomophthoromycetes	1	0.89	0.89	0.89
		Cr	Cryptomycota incertae sedis								2	0.29	-0.27	0.85
	unclassified													
